# When Extracellular Vesicles Go Viral: A Bird's Eye View

**DOI:** 10.20411/pai.v10i1.787

**Published:** 2025-02-14

**Authors:** Leonid B. Margolis, Yoel Sadovsky

**Affiliations:** 1 Faculty of Natural Sciences and Medicine, Ilia State University, Tbilisi, Georgia; 2 Magee-Womens Research Institute, Department of OBGYN and Reproductive Sciences, Microbiology and Molecular Genetics, University of Pittsburgh, Pittsburgh, Pennsylvania

**Keywords:** Extracellular vesicles, viruses, cell communication, cooperativity, antagonism

## Abstract

The science of extracellular vesicles (EVs) is a rapidly growing field that spans multiple aspects of normal physiology and pathophysiology. EVs play a critical role in most basic biological processes of cell-cell communications under normal conditions and in disease. EVs have “gone viral” not only in terms of research popularity, but also in our realization that they exhibit an elaborate crosstalk with viruses, particularly with the enveloped ones, which are also extracellular vesicles that are released by cells as a part of their virulence cycle yet are replicative. Here, we highlight some of the complexities underlying EV-virus crosstalk and pathways and provide our insights on key challenges from the viewpoint of EV biology.

## INTRODUCTION

Among various particles that are generated and released by cells in our body, 2 major types that have been thoroughly studied are extracellular vesicles (EVs) and viruses. While the importance of viruses, defined in Oxford Reference as “a minute particle that is capable of replication but only within living cells,” has been recognized for about 130 years [1], the relevance and impact of EVs, defined as the “lipid bilayer membrane-delimited, nano- to micro-sized particles that appear to be released by all cell types” [2], has been elucidated only relatively recently. EVs are now considered a central means of physiological or pathological communication among cells and organs within multicellular organisms, and even between organisms, such as maternal-fetal EV trafficking during pregnancy, passage in breastmilk lactation, host-microbiome EV interaction, and trafficking among plants and parasites [3–6].

The 3 best characterized EV types are (1) small EVs (exosomes) of approximately 30–150 nm in diameter that are formed as intraluminal vesicles within the cell's multivesicular bodies (MVB) and released to the extracellular space when the MVB fuses with the cell membrane; (2) membrane particles (ectosomes, microvesicles) of approximately 50–500 nm in diameter that are released by pinching off from plasma membranes; and (3) apoptotic bodies of the micron size, formed in the course of apoptotic cell disintegration. Although important, the latter are outside the scope of our current essay.

Mechanisms underlying the cargo loading, production, intracellular mobilization, release, and uptake are shared between EVs and many enveloped viruses. Both generally contain similar molecules, proteins, lipids, and nucleic acids, and both are targeted to particular cells and can affect their physiology. The ancient origin of EVs and viruses, which likely developed early in evolution as they are found in bacteria, plants, and algae, suggests that one is probably a descendent of the other, where viruses might have evolved from EVs, gaining the ability to replicate. Whereas divergent evolutionary trajectories cannot be excluded, the coexistence of EVs and viruses across hundreds of thousands of evolution years might have led multicellular organisms to usurp EVs as part of antiviral defense strategies and, in turn, led viruses to commandeer EVs to enhance their infectivity. Indeed, such reciprocal effects have been documented, surprisingly even for the same virus [7].

The field of EV biology has “gone viral” in the past 20 years, both in terms of its rapid spread to other domains of biology, biomedical diagnostics, and therapeutics, as well as by virtue of its complex interactions with viruses. However, progress in this field has been hampered by our limited understanding of basic mechanisms underlying EV function, both under normal conditions and in pathophysiological processes. Understanding this crosstalk is crucial for advancing our knowledge of EV function. In this short essay, we neither list various effects of EVs on viral infection, nor reprise recently reviewed assays on EV-related technologies, isolation methods, and EV applications to diagnostics or therapeutics [8–11]. Instead, we emphasize the complex basic problems regarding the interaction of EVs and viruses that must be solved in the field of EV biology in order to define the function of EVs as mediators of cell-cell communication in the context of viral infection.

**Figure 1.**
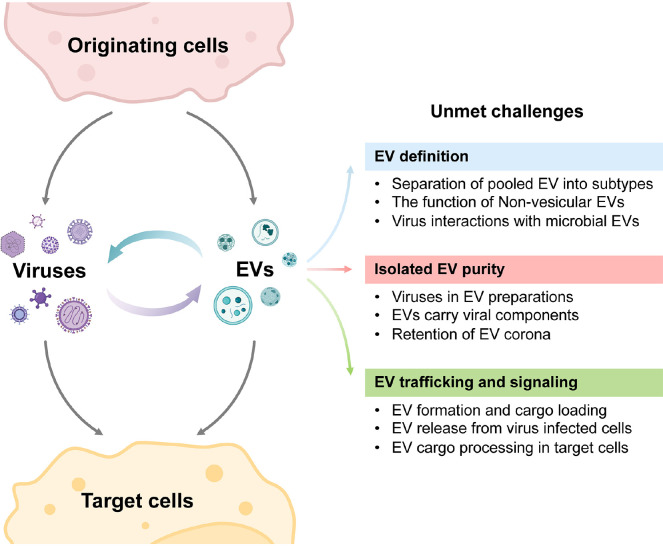
**Major challenges in our understanding of EV-virus interactions, depicting some of the complexities and key challenges from the viewpoint of EV biology.** The challenges shown on the right correspond to those noted in each of the text's sections. Created with BioRender.com.

## EVs AND VIRUSES: FRIENDS OR FOES?

The co-evolution of non-replicative EVs and replicative viruses resulted in intricate relationships between those particle types. These relationships are multifaceted, reflecting the diverse ways EVs and viruses can interact to either enhance or diminish viral spread. While numerous pathways underlying the effect of EVs on viral infection have been reviewed [12–14], this essay focuses on several striking examples of EV-virus interactions in order to highlight unmet challenges in understanding mechanisms underlying these relationships.

### EVs' Mimicry of Viruses

It is now well established that EVs generated from virus-infected cells carry viral elements (see [12, 13, 15]). These EVs are functionally similar to defective (noninfectious) viruses. Such EVs are commonly generated in quantities that exceed those of infectious virions [16]. Throughout the 20th century, the field of virology focused mostly on infectious viruses. The impact of viral components-laden EVs has only recently been recognized. For example, the HIV protein Nef, which plays an important role in HIV pathogenesis and comorbidities, is carried by EVs [17,18]. Other infection-promoting HIV components that are carried by EVs include HIV's cellular coreceptors CCR5 and CXCR4, which can increase cellular susceptibility to HIV infection [19]. Similarly, EVs with the viral transactivation response element (TAR) RNA promote cell survival and thus augment the release of virions [20,21]. In contrast, T cells release EVs that contain the HIV receptor CD4, which acts as a decoy to reduce the number of HIV viruses that can infect cells [7]. Hepatitis C virus (HCV)-infected cells also release EVs harboring the viral proteins E1 and E2 [22]. Similarly, SARS-CoV-2-infected cells release EVs that contain E and M viral membrane proteins [22]. EVs from infected cells may carry not only viral proteins but also viral RNA or DNA, and these EVs may shape immune response in target cells [23, 24]. Remarkably, even an entire virus (such as HAV or HCV) can be transferred into cells, cloaked inside EVs that provide an envelope to non-enveloped virions, and assist them to overcome the absence of specific viral receptors [25].

EVs carrying various viral molecules may affect not only the infected cells but also systemic immunity. For example, in non-human primates infected with Ebola virus, EVs carry a surface glycoprotein that attenuates immune response, in part, by depleting T cells, thus contributing to Ebola pathogenesis [16]. Also, Nef-containing EVs from HIV-infected cells, mentioned above, may attenuate T-cell response, thus reducing antiviral reactions [17]. In contrast, EVs released before viral spread can heighten antiviral cytokine responses by alerting the immune system via Toll-like receptor pathways [26–28].

### EVs May Affect Both Cells and Virions

EVs impacting viral infections can originate not only from infected cells but also from bystander cells and even bacteria. Bioactive EVs that carry viral elements are likely generated in many, if not all, viral infections. These EVs can affect target cells, including their susceptibility to viruses. However, we have limited mechanistic understanding of these phenomena, with only a few well-characterized examples. EVs with exposed phosphatidylserine (PS) occupy the target cell's membrane PS receptors and thereby decrease cell infectivity by Zika, West Nile, Chikungunya, and Ebola viruses, which use the PS receptor to infect cells [29, 30]. EVs can diminish viral replication in other cells by delivering specific miRNA [31, 32]. Interestingly, bacteria may also release EVs that shape target cell infectivity. For example, lactobacilli-generated EVs bind to HIV gp120 and decrease viral ability to bind to the host cell receptor [33]. In contrast, some EVs derived from *Porphyromonas gingivalis* bind to HIV to transfer the virus into nonpermissive cells where infection is established [5].

## EVs “GOING VIRAL” – CENTRAL CHALLENGES

Defining the key determinants that shape the interaction of EVs with viruses, whether cooperative or antagonistic, requires deeper understanding of EV biology. We must also ensure that EV-probing technologies do not create technical artifacts that are irrelevant to EV-virus crosstalk. In the section below, we define key investigative issues and their implications, all from the viewpoint of EV biology, and formulate a set of unmet challenges.

### EVs: Not Well Defined, Not Well Isolated

Despite their diversity, EVs remain poorly characterized compared to viruses. Methods for isolating EVs based on size and composition, as is done for viruses, are lacking. Currently the most popular techniques for EV isolation are ultracentrifugation and iodixanol (OptiPrep) gradient separation or size-exclusion chromatography. Ultracentrifugation isolates EVs of similar density, while size-exclusion chromatography isolates EVs of similar size irrespective of their biogenesis or chemical composition. However, both techniques ignore EV diversity, defined by other parameters. Immunoaffinity-based methods are another approach for isolating EVs (reviewed in [34]). Although initially it was more laborious and applied only to small quantities of EVs, high throughput immunoaffinity techniques are being developed [35]. Notably, immunoaffinity-based isolation methods target particular fractions of EVs on the basis of their specific antigens, which may be a limitation when the goal is to collect all EVs. Other currently developed methods, like asymmetric flow field-flow fractionation, also have limitations but are greatly needed to fully define the landscape of EV diversity [36–38].

Many published claims regarding EV purity are limited by inadequate analytical specificity and imperfect measurements. For example, EV size measurement oﬅen relies on the questionable assumption of a spherical EV structure, which is strongly influenced by the measurement technique. Nanoparticle tracking analysis and cryo-transmission electron microscopy provide different size distributions, varying not only in the range of EVs analyzed but also in the relative proportion of smaller to larger EVs [39]. Particle size estimation based on fluorescent probes depends on membrane-intercalating dyes, skewing results due to dye interaction with tissue-specific membrane lipid types. Even emerging single-vesicle technologies [40] may not accurately capture the full range of EV sizes. These factors may lead to an overestimation of EV size variability [41, 42].

The variability of EV lipid composition remains a challenge. Even approximate calculations suggest a vast number of possible EV lipid compositions. This number may exceed the total number of cells in a human body, which is on the order of 10^13^ [43]. Although the actual number may be lower due to limitations on phospholipid combinations and vesicle formation, the potential diversity of EVs remains vast. Moreover, current data suggest that the cell-specific EV-to-cell ratio spans 4 orders of magnitude, from 0.13 ± 0.1 erythrocyte-derived EVs/erythrocyte to (1.9 ± 1.3) × 10^3^ monocyte-derived EVs/monocyte [44]. Therefore, despite the EV potential diversity, it seems that different EVs may have a similar bioactivity, consistent with the commonly observed redundancy in most biological systems.

**Key Unmet Challenges in EV Characterization**

1. A major challenge in EV research is the lack of reliable markers to distinguish EVs of similar size. While viruses are oﬅen readily distinguishable, even aﬅer release, we lack reliable markers for EVs, particularly those in the 100–200 nm size range [45]. This lack of specific markers hinders our ability to separate EVs on the basis of their biogenesis pathways. Consequently, despite the likelihood that EVs from different origins play distinct roles in viral interactions and cell communication, many studies still rely on pooled EV fractions. This inability to separate EVs of similar physical characteristics but different biogenesis pathways is an important obstacle in studies on EV-virus interactions [46].
2. The dimensionality of communicating particles has been recently expanded by the discovery of *non-vesicular extracellular particles* (NVEPs), which in addition to the well-characterized lipoproteins, include exomeres, supermeres, and vaults. These NVEPs, which lack a lipid bilayer membrane, are present and oﬅen plentiful in the extracellular space and bodily fluids. Like EVs, they may participate in complex patterns of intercellular communications [2, 47, 48]. The overlapping size of many EVs and NVEPs make it difficult to separate them [47]. Moreover, EVs may physically interact with NVEP elements [49] to regulate their targeting and biological function, creating a poorly understood communication with target cells. These particles carry multidimensional information (molecular “voxels”) that can be integrated into a larger, unbiased landscape (EV/EP “hologram”) [50]. While individual effects of these particles, such as modulating target cell metabolism [51], are beginning to unfold, their interaction with viruses (or with other EV types) requires further research.
3. EVs are an essential part of microbiome bacteria communication with the host cells. Considering the importance of the microbiome for our physiology, further studies are needed to reveal the role of microbiome-generated EVs in viral infection.

### Purified EVs – Are They the EVs We Sought?

A key challenge in EV research is ensuring the purity of EV isolates. Knowledge about EV function is commonly based on *in vitro* experiments in which isolated EVs are incubated with different cellular and tissue cultures. To ensure physiological relevance, isolated EVs should accurately represent those found *in vivo*. Thus, assuring that EV isolation techniques do not result in technical artifacts that are irrelevant to EV-virus crosstalk becomes paramount. However, lipoproteins, like low-density lipoproteins (LDL), can co-isolate with EVs due to their similar size and density, and may even form complexes with EVs [49]. Consequently, some observed EV characteristics may actually be attributed to lipoproteins. Recently, methods have been developed to separate EVs from LDL [52,53], and they should be included in EV isolation protocols. Further, some EVs contain enzymes [54, 55] that may modify the EV cargo over time. Thus, inferring from *in vitro* studies to physiologically relevant *in vivo* systems requires additional validation.

EVs, like other colloidal particles, can acquire a corona (crown), a halo of adsorbed molecules on their surface. The concept of an EV corona of attached proteins was recently introduced by Buzas et al [56]. As EVs circulate in protein-rich biofluids, they also acquire coronas. Importantly, various proteins that form coronas and that might be critical for EV function are attached to the EV surface with variable affinity. Some corona proteins bind tightly to EVs and resist removal, even with rigorous purification [56, 57], while others are loosely associated and readily lost during isolation. Yet, these proteins may define some EV functions. For example, these corona proteins may enter target cells even without internalization of their host vesicles or may signal through cell surface receptors [57].

Several technologies have been proposed to elucidate the role of corona components, including gentle digestion of surface components, permeabilizations, physical disruption of the corona, or reconstruction of the coronas with specific properties [58]. The presence of many of these proteins in the common medium bathing various EVs may lead to apparent EV redundancy, masking some of the EV heterogenicity discussed above. Thus, direct studies of corona structure and function are critical for full understanding of EV-mediated signaling.

**Key Unmet Challenges in EV Purification**

1. Isolating pure EVs is crucial for EV research. Most EV isolation methods that separate EV fractions by size or density may contain viral particles. Although progress has been made in developing purer EV preparations [59], this remains a challenge, particularly for retroviruses, which share similar size, density, and other biophysical properties with many EV subtypes [15]. Reliable techniques to isolate “pure” EVs need to be developed.
2. EVs generated by retrovirus-infected cells commonly carry viral proteins or even fragments of the viral genomes. These EVs can be classified as noninfectious viruses or viral particles. They interact with cells, triggering responses similar to those induced by enveloped viruses. This similarity adds complexity to functional studies, which must account for these EV-mediated effects.
3. Both EVs and viral particles harbor a corona [60]. Interactions between EV and viral coronas may influence their crosstalk. Consequently, loss of the corona during EV isolation and purification may render studies on EV-virion interactions less reflective of their functional interaction *in vivo*, as the corona may mediate or modulate these interactions.

### The Intricacies of EV Trafficking and Signaling

For multicellular organisms to function properly, coordinated communication between cells, tissues, and organs is essential. Such interaction pathways include direct membrane-contact interactions or cytoplasmic bridges between adjacent cells, released signaling molecules (eg, proteins, lipids, hormones, cytokines), and communication via EVs. Each type of communication has its own advantages and limitations. What are the advantages of EVs in this role?

EVs are a key component of the language cells use to communicate [61, 62]. Akin to human languages, where the first few words in a sentence suggest what the full sentence implies, discrete EV molecular cues may reveal the full composition of each EV. For example, EV envelope composition may disclose the general type of cargo molecules even prior to intracellular endocytic processing and activate particular intracellular pathways for cargo processing even prior to EV entry into cell cytoplasm [63, 64]. Packaging of communication signals likely protects the EV cargo against degradation in the plasma or other extracellular fluids. Discrete molecules on the EV surface may serve as a QR code that provides precise targeting in highly complex systems. Packaging multiple cargo components within a single EV may provide synergistic benefits. Lastly, EVs may also participate in homeostatic feedback mechanisms, responding to cues from hormones, growth factors, metabolites, or even to signals from other circulating EVs and may affect the biology of nearby or distant tissues.

Messaging through EVs requires complex signal processing in recipient cells, more so than processing single molecules. EVs interact with recipient cells through binding at the cell surface, signaling to cell-surface receptors by the EV surface molecules, cytoplasmic EV uptake or endocytosis, and trafficking of EV components into diverse intracellular compartments, with cargo shuttling and processing in the proper organelles [61, 64–69]. A key hurdle in investigating the mechanisms of these processes is the need for multiple different techniques to capture these multi-stage processes. To fully understand these processes, innovative approaches to block specific intracellular components are critically needed to establish discrete pathways.

Understanding the biological meaning of EV diversity in cell communication requires addressing several unresolved questions related to EV biogenesis, release from cells, and transfer to target cells. Deciphering the full intricacies of EV biogenesis is challenging [61, 70, 71], although the formation of intraluminal vesicles through the action of endosomal sorting complexes required for transport (ESCRT), syntenin-ALIX, tetraspanins, or other cytoskeleton machineries within microdomains may be similar for multivesicular endosomes and the cell membrane [61]. Such resemblance hinders understanding of the unique aspects of biogenesis of diverse EVs and makes the probing of individual components more complex. Similarly, cellular pathways underlying the formation of intracellular endosomes and EVs overlap. What determines the fate of a vesicle or a multivesicular endosome to become an endocytic actor, to be shuttled to lysosomes, or to be a released, secretory vesicle? Another intriguing concept is the role of selective cargo in modulating EV biogenesis pathways [72]. Do key determinants include the type of cargo (membrane-bound or not) or the cargo's biochemical composition? Are there also subpopulations of multivesicular endosomes, and how do they interact with other cellular organelles? What is the impact of the cell membrane location in polarized cells on EV release [73]?

Several variables influence EV trafficking in the circulation. The release of EVs from the apical or basolateral surface of polarized cells is asymmetrical, and largely dependent on different machineries [73–78]. It is currently unknown whether the release from the apical or basolateral surfaces of polarized cells affects EV dynamics and interaction with viruses in extracellular space. Similarly, the effect of glycocalyx and other extracellular matrices on EV movement in the extracellular space, and the role of EVs' biophysical properties in determining EV release and trafficking require further investigation. While EVs can be found in any biological fluid, what determines their half-life in that fluid? Further, EVs are known to cross bodily barriers, such as the blood capillary endothelial cells, lymphatic cells, or the blood-brain barrier [79], but the mechanisms underlying these processes and their effect on EV function remain to be uncovered [72].

Despite our knowledge of several endocytic pathways mediating EV uptake, many aspects of cell targeting and the intercellular processing of EV cargo signals are unclear and may be cell-type specific [80]. Are different EV subtypes processed differently? Does the EV corona (see above) play a role in directing EVs to target cells? Once an EV is expected to be inside a cell, it is commonly assumed that any effect on target tissue or cells *in vivo* or *in vitro* can be attributed to EV cargoes. Yet, the internalized EVs in target cells maybe targeted for degradation in lysosomes. What guides the endosomal escape of EV cargo prior to degradation in lysosomes within target cells? In this regard, an overlooked aspect is the relative advantage of EVs compared to other communication means, such as cell-cell contact, nanotubes or soluble factors like cytokines, or extracellular RNA.

**Key Unmet Challenges in Mapping EV Trafficking**

1. Both EVs and viruses utilize the ESCRT system for their formation. However, these biogenesis pathways, which are likely essential to defining EV biology, are not currently used to differentiate EV subtypes. A better understanding of EV formation and cargo loading during biogenesis will improve our understanding of EV-virus crosstalk. While some EVs passively reflect cytoplasmic content, others actively sort molecules, such as RNA, into their cargo [81, 82]. More insights into EV biogenesis are needed to shed light on the EV-virus interaction.
2. A key hurdle in investigating the mechanisms of EV membrane binding and endocytosis is the need for several different techniques to capture these multi-stage processes. To establish discrete pathways, innovative approaches are needed to block specific intracellular components. Understanding these mechanisms is crucial for developing targeted interventions.
3. EVs, like many viruses, can enter target cells through various mechanisms, including fusion and endocytosis. The role of membrane binding and endosomal processing in determining the interaction of EVs and viruses in target cells remains poorly understood. Is endosomal escape by viruses affected by the presence of EV surface proteins and cargo? Does such interaction functionally influence target cell pathogen recognition receptors and other cellular antiviral response pathways? How are viral genomic elements, carried by EVs, processed inside the target cells, and how do they change cell physiology?

## CONCLUSIONS

Addressing the main challenges discussed in our perspective has direct implications for clinical medicine. Our grasp of disease pathogenesis will be markedly improved by better insights into EV trafficking. A key example is the role of EVs in carcinogenesis and the metastatic spread of a primary tumor, where trafficking of tumor-derived EVs may play a major role in shaping the tumor microenvironment and the formation of distant pre-metastatic niches (reviewed in [83]). Additionally, improved EV isolation technology may allow the identification of virus-containing EVs, which will serve both diagnostic and therapeutic targeting of virion-containing EVs. Furthermore, the effect of EVs on the immune system may bolster research into their use in modulating antiviral immune responses and other pathways underlying viral resistance [84]. These advancements necessitate better technologies for EV characterization and standardization of quality and reporting, which are essential for improved diagnostics, disease prediction, and prognostics [2, 47]. This also includes the characterization of NVEPs, such as supermeres, where analysis of their cargo molecules suggests a role for these particles in common diseases like Alzheimer's, cancer, and cardiovascular disorders [51]. Further, as the EV corona harbors not only proteins but also lipoproteins and nucleic acids, it may play a critical role in EV distribution and the targeting of engineered EVs for therapeutic purposes [85, 86]. It may also affect the ability of EVs to cross barriers like the blood-brain barrier. Therapeutically, these parameters are particularly important when Good Manufacturing Practices guidelines must be met to ensure quality and safety before introduction into clinical care.

Given the burgeoning field of EV research, the key roles of EVs in communication among cells and organs in health and disease, in particular the infectious ones, are gradually being elucidated. However, understanding specific effects of EVs in viral infection remains hampered by the complexity of the EV landscape in terms of their structure, size, density, formation, cargo molecules, loading pathways, mechanism of uptake, cargo sorting in target cells, and biological function [87]. To further our understanding of the complex role of EVs in cell-cell communications under normal conditions and in viral infection, we need to find reliable markers that distinguish EVs on the basis of their release pathways, under standard laboratory conditions that faithfully capture processes that occur *in vivo*. Specific mechanisms, such as the loading of EV cargoes and the integration of membrane proteins into EVs, in particular into the virally encoded ones, warrant investigation. We need to investigate the mechanisms of EVs' interactions with viruses and whether these occur both inside and outside the cells and define the role of the various EV coronas in EV bioactivity and in their interaction with viruses.

To address these issues, a systems EV biology, rooted in statistical and computational methods, might be needed to provide holistic insights into the complex interactions among cell of origin, assorted vesicular messages, and recipient targets. Realizing these complexities forces us to re-think EV types, their redundancy, communication language, and intriguingly, EV homology to viruses and their crosstalk. Understanding how this general system is organized may require the involvement of virology, cell biology, and broad expertise in systems biology, bioinformatics, even in structural linguistics, as EVs constitute a language by which cells communicate to each other [61, 62]. Such interdisciplinary collaboration may constitute the next frontier of EV science.
